# Hydrophobic Mycobacterial Antigens Elicit Polyfunctional T Cells in *Mycobacterium bovis* Immunized Cattle: Association With Protection Against Challenge?

**DOI:** 10.3389/fimmu.2020.588180

**Published:** 2020-11-12

**Authors:** Lindert Benedictus, Sabine Steinbach, Thomas Holder, Douwe Bakker, Christina Vrettou, W. Ivan Morrison, Martin Vordermeier, Timothy Connelley

**Affiliations:** ^1^ Division of Infection and Immunity, The Roslin Institute, The University of Edinburgh, Easter Bush, United Kingdom; ^2^ Department of Bacteriology, Animal and Plant Health Agency, Weybridge, United Kingdom; ^3^ Independent Researcher and Technical Consultant, Lelystad, Netherlands; ^4^ Centre for Bovine Tuberculosis, Institute for Biological, Environmental and Rural Sciences, University of Aberystwyth, Aberystwyth, United Kingdom

**Keywords:** bovine tuberculosis, lipopeptides, *Mycobacterium bovis*, vaccine, hydrophobic antigens

## Abstract

Bovine tuberculosis (bTB), caused by *Mycobacterium bovis*, is a chronic disease of cattle with a detrimental impact on food quality and production. Research on bTB vaccines has predominantly been focused on proteinaceous antigens. However, mycobacteria have a thick and intricate lipid outer layer and lipids as well as lipopeptides are important for immune-evasion and virulence. In humans, lipid extracts of *M. tuberculosis* have been shown to elicit immune responses effective against *M. tuberculosis* *in vitro*. Chloroform-methanol extraction (CME) was applied to *M. bovis* BCG to obtain a hydrophobic antigen extract (CMEbcg) containing lipids and lipopeptides. CMEbcg stimulated IFN-γ^+^IL-2^+^ and IL-17A^+^IL-22^+^ polyfunctional T cells and elicited T cell responses with a Th1 and Th17 cytokine release profile in both *M. bovis* BCG vaccinated and *M. bovis* challenged calves. Lipopeptides were shown to be the immunodominant antigens in CMEbcg, stimulating CD4 T cells *via* MHC class II. CMEbcg expanded T cells killed CMEbcg loaded monocytes and the CMEbcg-specific CD3 T cell proliferative response following *M. bovis* BCG vaccination was the best predictor for reduced pathology following challenge with *M. bovis*. Although the high predictive value of CMEbcg-specific immune responses does not confirm a causal relationship with protection against *M. bovis* challenge, when taking into account the *in vitro* antimycobacterial phenotype of CMEbcg-specific T cells (e.g. Th1/Th17 cytokine profile), it is indicative that CMEbcg-specific immune responses could play a functional role in immunity against *M. bovis*. Based on these findings we conclude that lipopeptides of *M. bovis* are potential novel subunit vaccine candidates and that further studies into the functional characterization of lipopeptide-specific immune responses together with their role in protection against bovine tuberculosis are warranted.

## Introduction

Bovine tuberculosis (bTB) has a huge impact on animal and human health and additional control strategies to curtail the impact of bTB are needed (1, 2). The global prevalence of bTB is estimated to be as high as 9% (1) and costs of the disease are predicted to be $3 billion annually for the cattle industry alone (3). *Mycobacterium bovis* is the predominant cause of bTB, but all members of the *Mycobacterium tuberculosis* complex are thought to be able to cause tuberculosis in cattle (4, 5). *Mycobacterium bovis* has a broad host range and is an important zoonotic agent. In spite of the fact that in many countries the public health risk of bTB has been alleviated by the pasteurization of milk, *M. bovis* is estimated to cause 121,000 zoonotic TB cases annually, predominantly in low- and middle-income countries (2, 6). *Mycobacterium bovis* shares a 99.95% sequence identity to *M. tuberculosis* (5) and the pathogenesis of bTB is similar to tuberculosis in humans (7). Bovine TB has been eradicated or is under control in a large number of high-income countries, following the implementation of disease control programs based on a test and cull approach in combination with strict movement controls (8, 9). However, eradication of bTB has proven difficult in several countries, particularly when there is spill-over from bTB reservoirs in wildlife (10–12). Additionally, implementation of test-and-cull programs may not be socially or economically acceptable, particularly in many low- and middle-income countries (13, 14). In these situations, vaccination against *M. bovis* could be an alternative control strategy. Bacille Calmette-Guérin (BCG), a live attenuated strain of *M. bovis*, has been used as a vaccine since the 1920’s and, despite decades of research, is still the only available vaccine against tuberculosis in humans and livestock (1, 15). However, BCG vaccination provides variable protection in cattle [reviewed by Waters, Palmer (3), Buddle, Vordermeier (16)] and, importantly, because BCG vaccination interferes with bTB diagnostics that are based on the use of the tuberculin skin test (17–19), it is incompatible with most bTB disease control programs. There is, therefore, an urgent need for novel vaccines against bTB that do not interfere with routine bTB diagnostics.

Numerous TB vaccine candidates have been tested or are being developed, including variations upon BCG (different vaccination regiments, genetic modification), other (attenuated and/or genetically modified) mycobacteria either live or dead, and (vectored) sub-unit vaccines [reviewed for cattle by Vordermeier, Jones (1) and for humans by Khoshnood, Heidary (15)]. Well-known examples of sub-unit vaccines are those based on the immunodominant, secreted, protein antigens Ag85, ESAT6, and CFP10, some of which have been used in experimental trials in cattle and are in various phases of clinical trials in humans. Almost all of this research has in common that it is focused on the use of protein antigens, either because (secreted) proteins are used as vaccine antigen or because protein antigens are used as readouts to measure immune responses following vaccination with whole mycobacteria. However, mycobacteria have a thick and intricate lipid outer layer and lipids and lipoproteins play a role in immune-evasion and virulence (20, 21). Interestingly, these cell wall associated hydrophobic molecules have been shown to be potent antigens in humans. Several lipids of *M. tuberculosis* are recognized by CD1 restricted T cells (22–26) and a substantial proportion of the *M. tuberculosis*-specific T cells in TB infected humans target lipopeptides and have antimycobacterial effector functions (27, 28). Hydrophobic and cell wall-associated antigens have been largely overlooked in previous antigen screens for *M. bovis* and there is a paucity of data on immunity against lipid antigens in bTB. Van Rhijn, Nguyen (29) showed that T cells from bTB infected cattle respond to a lipid extract of *M. bovis* and phosphatidylinositol mannosides, a class of mycobacterial glycolipids, were shown to elicit an immune response in *M. bovis-*infected cattle (30). Exploring non-conventional antigens, such as hydrophobic lipopeptides and lipids, has several advantages for vaccine design: i) the potential vaccine antigen repertoire is expanded and diversified; ii) some hydrophobic antigens have intrinsic adjuvant properties (e.g. TLR activation by lipopeptides); iii) lipid antigens can be more conserved because they are not the product of a single gene and therefore have lower mutation rates; and finally, iv) current diagnostics of bTB are based on protein antigens or protein extracts and therefore vaccines based on lipid antigens are not predicted to interfere with bTB diagnostics.

The aim of the current study was to further our understanding of immune responses against hydrophobic antigens in bovine tuberculosis and to identify potential novel vaccine antigens. T cell responses against hydrophobic antigens of *Mycobacterium bovis* BCG were characterized both in BCG vaccinated and bTB infected animals. We found that hydrophobic antigens, extracted from BCG using chloroform-methanol, elicited polyclonal T cell responses in *M. bovis* immune animals. Our data suggest that lipopeptides, presented to CD4^+^ T cells *via* MHC class II, were the immunodominant antigens in this extract and that immune responses against the hydrophobic antigen extract following BCG vaccination was a predictor for reduced pathology following *M. bovis* challenge.

## Materials and Methods

### Cattle and Sample Collection

All animals were housed at the Animal and Plant Health Agency at the time of blood sampling, and procedures were conducted within the limits of a United Kingdom Home office license under the Animal (Scientific Procedures) Act 1986, which were approved by the APHA Animal Welfare and Ethical Review Body (AWERB) committee.

Twenty-four 6-months old male Holstein-Friesian or Holstein-Friesian cross calves were acquired from a TB-free herd located in a non-endemic area and selected for negative test results for the whole blood stimulation IFN-γ release assay for a BCG-vaccination and *M. bovis* challenge study. Six calves served as control animals and 18 calves were vaccinated with 0.5 ml of 4.6 × 10^6^ CFU BCG Danish SSI 1331 (Statens Serum Institute) *via* the subcutaneous route. In-house grown BCG Danish SSI 1331, stored frozen in aliquots, was thawed on the day of vaccination and diluted with 7H9 broth (Becton Dickinson) with 0.05% Tween 80 (Sigma). The actual vaccination dose was confirmed retrospectively by culture. Animals were randomly allocated to the two groups by the lottery principle. Nine weeks following vaccination, all 24 animals were infected with 10^4^ CFU *M. bovis* AF2122/97 *via* the endobronchial route. For this phase, a randomized block design was applied with one control and three BCG vaccinated animals randomly allocated to one animal room. The challenge inoculum was prepared from a frozen stock and the challenge dose was confirmed retrospectively by culture. The inoculum was administered just before the tracheobronchial-junction using an endoscope. Eleven weeks post *M. bovis* challenge (20 weeks post BCG vaccination) animals were euthanized and post-mortem examination was performed as described in detail elsewhere (31). In summary, lungs and lymph-nodes of head and pulmonary regions were examined for the presence of gross visible lesions. The severity of the gross pathology of lungs and lymph nodes were scored using a semi-quantitative scoring system and combined resulting in a total pathology score (31). Blood samples were taken at regular intervals to monitor immune responses. Additional blood samples were obtained from naturally *M. bovis*-infected cattle from British herds with known bovine tuberculosis outbreaks, which were positive for the single intradermal comparative cervical tuberculin skin test and the whole blood antigen stimulation IFN-γ release assay.

### PBMC Isolation and Culture

Peripheral blood mononuclear cells (PBMC) were isolated from blood by density gradient centrifugation using Histopaque-1077 (Sigma-Aldrich) and cryopreserved or used directly. For antigen stimulation, PBMC were seeded in triplicate in 96-well U-bottom plates at 4 × 10^5^ cells/well in 200 µl cell culture medium (RPMI 1640 containing 25 mM HEPES, 5 × 10^−5^M β-mercaptoethanol, 100 U/ml penicillin, 100 μg/ml streptomycin, ± 0.1 mM NEAA, 2 mM Glutamine/GlutaMax, all from Gibco Life Technologies, and 10% fetal calf serum from Sigma) in the presence of relevant antigens, unless indicated otherwise for specific methods. For all experiments, cells or blood were incubated at 37°C in 5% CO_2_.

### Antigens and Antibodies

Hydrophobic antigens were extracted from *M. bovis* BCG Danish 1331 using a chloroform-methanol extraction method modified from Layre, Paepe (32). Lyophilized bacteria were resuspended in PBS and washed twice with PBS by centrifuging (15,000 g, 2 min) and removal of the supernatant. Clean glass pipettes, borosilicate glassware and PTFE lined caps (all Corning/VWR) were used for all subsequent procedures involving solvents. The bacterial cell pellet was resuspended in a 20 times pellet volume of CHCl_3_:CH_3_OH (2:1, v/v) and incubated for 2 h at RT on a rocker. After centrifugation (4,000 g, 10 min), the liquid phase was collected and the bacterial pellet was subjected to subsequent extractions using CHCl_3_:CH_3_OH (1:1, v/v) and CHCl_3_:CH_3_OH (1:2, v/v) for 1 h at RT, respectively. The combined supernatant of all three extracts was centrifuged and the supernatant collected in a new tube. The extract was subjected to a Folch wash by the addition of 20% of the total extraction volume of 0.9% NaCl and vortexing the solution. Following centrifugation the upper phase (the Folch wash) was removed, the sample centrifuged again, and the remaining Folch wash was removed. The CHCl_3_:CH_3_OH phase was filtered through a 0.2 µM PTFE filter (Millipore) into a clean weighed tube. The sample was dried under nitrogen while heated to 40°C and the dry mass was weighed. The chloroform-methanol extract of BCG (CMEbcg) was dissolved in CHCl_3_:CH_3_OH (1:1, v/v) at 4 mg/ml and stored at −20/−80°C. Prior to use in experiments, an aliquot was dried under nitrogen or using a heating block (40–50°C) only and resuspended in appropriate media followed by sonication for 10 min in a heated water bath at 37–50°C, brief vortexing, incubation at 50°C for 50 min, brief vortexing and again 10 min sonication at 37–50°C and used at a final concentration of 20 µg/ml to stimulate cells. Bovine and Avian tuberculin purified protein derivative (PPDB and PPDA, Prionics) were used at a final concentration of 300 IU/ml and 250 IU/ml respectively. Recombinant 6 kDa early secretory antigen (ESAT6, Lionex) and 10 kDa culture filtrate protein (CFP10, Lionex) were used at 2.5 µg/ml. A pooled peptide cocktail of Antigen 85a (Ag85a, Mimotopes) consisting of peptides of 15 amino acids in length overlapping by ten residues was used at 20 µg/ml. Concanavalin A (Sigma) was used at 2.5 µg/ml and PWM (Sigma) at 10 µg/ml.

The following antibodies were used: anti-CD3 (MM1a), anti-CD4 (ILA12), anti-CD8 (ILA51), anti-TCRδ (GB21a), anti-CD25 (ILA111), anti-CD45-RO (ILA116), anti-CD172a (ILA24), anti-DRB3 (ILA21), anti-DQ (CC158), anti-CD1b1/3 [bCD1b3 (33)], IgG2a isotype (Av37) all produced in-house; anti-CD25 (CACT108a), anti-CD4 (CC8), anti-DRB3 (TH14B), and anti-DQ (TH81A5) from Washington State University Monoclonal Antibody Center; anti-CD4 (CC30), anti-CD14 (Tük4), polyclonal goat anti-huIgG F(ab’)_2_-FITC from Bio-Rad; anti-CCR7 (3D12) from eBioscience; streptavidin-PE from Biolegend or BD Pharmingen; anti-perforin (dG9) from BD Biosciences; anti-granulysin (rabbit polyclonal biotinylated IgG) from Raybiotech; rabbit polyclonal biotinylated IgG isotype from Abcam; mouse IgG1 and IgG2a isotype controls and polyclonal goat anti-muIgG2b-AF633 from ThermoFisher Scientific; anti-boTCRδ (GB21A) and (biotinylated) polyclonal rabbit anti-boIL-17A (Kingfisher Biotech); recombinant bivalent anti-boIL-22 Fab (34). For all flow cytometry experiments, antibodies were either directly conjugated with fluorochromes in house (Pacific Blue™, Alexa Fluor™ 488, and Alexa Fluor™ 647 antibody labeling kit, Invitrogen), bought directly conjugated or labeled with isotype-specific fluorochrome-conjugated antibodies. To exclude dead cells, cells were stained with Fixable Yellow or Violet Dead Cell Stain (ThermoFisher Scientific) in PBS. Cells were stained at predetermined optimal antibody concentrations in PBS buffer supplemented with 2% FCS and 0.01% Azide at 4°C for 15–30 min. The same buffer was used for washing. Samples were acquired on a LSRFortessa™ (BD Biosciences) or CyAn ADP analyzer equipped with 405, 488, and 642 nm lasers (Beckman Coulter) for intracellular flow cytometry. Data were analyzed using FlowJo software (TreeStar Inc.). Flow cytometry gating strategy is outlined in **Supplemental Figure 1**.

### Two-Dimensional Thin Liquid Chromatography

The lipid content of CMEbcg was analyzed using two-dimensional thin liquid chromatography (2D-TLC) according to the method described by Dobson, Minnikin (35). CMEbcg was spotted unto aluminum backed silica gel 60 F254 TLC plates (Fisher Scientific). Plates were dried (10 min 80°C) and placed in the appropriate direction in equilibrated TLC tanks containing solvents mixtures as detailed in **Table 1**. Between each run and before staining, plates were dried (10 min 80°C). For staining, plates were sprayed with molybdophosphoric acid (5% in 95% ethanol, Sigma-Aldrich) and subsequently charred with a hot air gun. Lipid classes were identified by comparing the staining pattern to previously published 2D-TLC analyses of mycobacterial lipid extracts (35, 36).

**Table 1:** Solvents for 2D-TLC characterization of mycobacterial lipids.

Solventsystem	Rundirection	Solvents (v)	Runs	Polarity
**A**	1	petroleum ether : ethyl acetate (98:2)	3	Apolar
	2	petroleum ether : acetone (98:2)	1	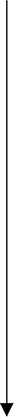
**C**	1	petroleum ether : acetone (92:8)	1	
	2	toluene : acetone (95:5)	1	
**D**	1	chloroform : methanol : water (100:14:0.8)	1	
	2	chloroform : acetone : methanol : water (50:60:2.5:3)	1	
**E**	1	chloroform : methanol : water (60:30:6)	1	Polar
	2	chloroform : acetic acid : methanol : water (40:25:3:6)	1	

### SDS-PAGE

CMEbcg was dissolved in Laemmli buffer by sonication and heating as described for medium. Antigens were incubated at 95°C for 5 min in Laemmli buffer. Samples or Precision Plus Protein Standards (Bio-Rad) were loaded onto a 14% Tris-Glycine gel (Novex) and run under denaturating conditions. Proteins were visualized using GelCode™ Blue Safe Protein Stain (ThermoFisher Scientific).

### Proliferation Assays

To measure proliferation, PBMC were stained with CellTrace Violet at 1.75 µM (Invitrogen) in Hanks Balanced Salt Solution (Gibco Life Technologies) for 10 min at RT before antigen stimulation. Staining was quenched by adding cell culture medium and incubating for 10 min at RT. Cell proliferation was assessed by flow cytometry as the percentage of CTV low cells (**Supplemental Figure 1**). Antigen specific proliferation was obtained by subtracting medium control values (spontaneous proliferation) from the antigen stimulation values and are indicated as Δ % CTV low.

### Cytokine Measurements

Five days following stimulation of PBMC with antigen, as described above, tissue culture plates were centrifuged (300 g for 10 min) and supernatants were harvested and stored at −80°C before use. IFN-γ was measured using the BOVIGAM ELISA (Prionics) according to the manufacturer’s instructions, with addition of a recombinant IFN-γ (ThermoFisher Scientific) standard curve. IL-17A was measured as described for bovine CXCL10 (37) using polyclonal rabbit anti-boIL-17A (1.25 µg/ml), biotinylated polyclonal rabbit anti-boIL-17A (1.0 µg/ml), and recombinant boIL-17A (all from Kingfisher Biotech). IL-22 was measured by an in-house ELISA using recombinant bivalent anti-boIL-22 HuCAL antibodies (34) and custom polyclonal anti-boIL-22 antibodies (Abcore). A Multiskan Ascent (Thermo Labsystems) plate reader was used to measure the absorbance at 450 nm.

TNF-α, IL-1β, IL-12 and IL-10 were simultaneously detected using the MSD multiplex platform (Meso Scale Discovery) as previously described (38). In summary, MSD multiplex 96-well plates were supplied pre-coated with capture antibodies on spatially separated locations. Blocking, incubation of samples or recombinant cytokines, washing, and addition of detection antibodies were similar to a conventional ELISA. Detection antibodies were SULFO-TAG™ (Meso Scale Discovery) labeled and emit light upon electrochemical stimulation initiated at the electrode surface of the MSD-plates and the luminescent signal for each cytokine at the defined spatial locations was measured on a MSD-6000. For IFN-γ, TNF-α, IL-1β, IL-12, and IL-10 samples were measured in duplicate. The IL-17A and IL-22 ELISA showed a high precision and reproducibility and samples were measured as singletons. Duplicate samples of the standard curve were checked for each ELISA plate to confirm that the variation for each ELISA plate was low.

Heparinized whole blood samples from the calves in the BCG-vaccination *M. bovis* challenge study (1.5 ml in 24-well plates) were stimulated with PPDA and PPDB within 6 h of drawing blood and incubated for 20–24 h. Tissue culture plates were centrifuged (300 g for 10 min) and supernatant harvested and stored at −80°C before measuring IFN-γ by ELISA as described above, except that ΔOD PPDB-PPDA values, rather than IFN-γ concentrations, were calculated.

### Proteinase K Digestion

Antigens, dissolved or diluted in RPMI as described, were treated with 400 µg/ml Proteinase K (ThermoFisher Scientific) for 60 min at 50°C in a waterbath. Proteinase K was subsequently inactivated by denaturating for 10 min at 85°C. For mock treatment, the proteinase K was denatured before addition to the samples. An equal amount of cell culture medium with all additives at double concentration was added and the CMEbcg antigens were subjected to another round of dissolving in order to dissolve any lipid antigens that were not dissolved in the original RPMI only buffer.

### Sodium Hydroxide Delipidation

Antigens, dissolved or diluted in RPMI as described, were treated with 0.12 or 0.22M sodium hydroxide for 30 min at 50°C in a waterbath, followed by neutralization of the sodium hydroxide with an equimolar amount of hydrochloric acid to restore the pH to ~7.2. For mock treatment, sodium hydroxide and hydrochloric acid were added at equimolar amounts in the first step. An equal amount of cell culture medium with all additives at double concentration was added and the CMEbcg antigens were subjected to another round of dissolving.

### Blocking of Antigen Presentation

Antibodies blocking antigen presentation *via* DR (ILA21, TH14B), DQ (CC158, TH81A5), and CD1b (bCD1b3) or relevant isotype control antibodies were added to PBMC and incubated for 30 min at 37°C 5% CO_2_ before addition of antigens. Final antibody concentration was 10 µg/ml.

### *In Vitro* T Cell Expansion

To investigate antigen-specific T cell responses, T cells were expanded *in vitro* and re-stimulated with antigens. PBMC were seeded at 2–3 × 10^6^ cells/ml in 0.5 ml or 1 ml cell culture media in 48-well or 24-well plates with PPDB, CMEbcg, or Ag85a.

#### T Cell Activation

To analyze T cell activation following antigen re-stimulation, PBMC were cultured for 10 days. The medium was partially replaced and supplemented with 40 IU/ml recombinant human IL-2 (Chyron) on day 4. On day 6/7 half of the medium was replaced with normal cell culture medium. T cells were harvested on day 10, counted and re-stimulated with antigen and irradiated autologous *Theileria annulata*-transformed cells (TaA) as antigen presenting cells (39) at a 1:2 ratio of TaA:T cells. Three days following re-stimulation CD25 expression and blasting of T cells (high forward and sideward scatter) was measured by flow cytometry.

#### Cytotoxicity Assays

For the cytotoxicity assays, cells were cultured for 13 days and supplemented with 20 IU/ml IL-2 at day 4 and 7 and the medium was partially replaced on day 10 and 12. On day 13 T cells were harvested, counted, and added to CellTrace Violet labeled autologous monocytes seeded at 3 × 10^4^ cells/well in 96-well plates in the presence of antigen. Monocytes were isolated from cryopreserved PBMC by labeling with monoclonal antibody ILA24 followed by isolation using Mouse IgGspecific paramagnetic beads (Miltenyi) according to the manufacturer’s instruction. Following a 20 h incubation period cells were washed with PBS and put on ice for 15 min to detach the adherent cells. Cells were then resuspended by pipetting, washed with PBS, pelleted, and stained with Fixable Yellow Dead Cell Stain. Precision count beads (1 × 10^4^ beads/well, BioLegend) were added and the samples analyzed by flow cytometry. Monocytes were identified as CellTrace Violet positive cells and the viability dye and the counting beads allowed enumeration of the percentage and absolute number of live cells per well. Specific lysis was calculated by expressing the results relative to the average value for the medium control condition for all animals according to the following equation: % specific lysis = (average medium control – sample)/average medium control × 100. The expression of perforin and granulysin by T cells was also analyzed on day 13. First, harvested T cells were stained with T cell phenotyping markers, followed by fixation of the cells in a 2% formaldehyde buffer and permeabilization using FACS Permeabilizing Solution 2 (BD bioscience). Intracellular granulysin and perforin were stained in the presence of permeabilizing solution using the mouse-anti-human perforin mAb dG9 (BD bioscience, isotype included) and polyclonal biotinylated rabbit anti-bovine granulysin antibodies (Raybiotech, isotype control from Abcam), respectively, using a two-step intracellular staining protocol.

#### Cultured IFN-γ/IL-2 FluoroSpot Assay

Cryopreserved PBMC were cultured at 2 × 10^6^ cells/ml in the presence of 300 IU/ml PPDB or 10 µg/ml CMEbcg. On day 4 and 7 cells were fed with cell culture medium supplemented with recombinant human IL-2 (Gentaur) at a final concentration of 10 IU/ml. Where necessary, cells were fed again between day 7 and 9. On days 10 and 12, half of the medium was replaced with IL-2-free medium. Antigen expanded cells were harvested and washed on day 13 for FluoroSpot assays and intracellular cytokine staining. Monocytes were isolated from cryopreserved PBMC using CD14-specific paramagnetic beads (Tük4, Miltenyi) according to the manufacturer’s instruction and incubated with 300 IU/ml PPDB or 10 µg/ml CMEbcg at 37°C and 5% CO_2_. For the FluoroSpot, monocytes (5 × 10^3^/well) were cultured directly on antibody coated PVDF filter plates (MultiscreenHTS IP-FL, Merck Millipore, UK) overnight before adding 1 × 10^4^ day 13 cell blasts to each of the wells with antigen loaded monocytes. Coating and development of the IFN-γ/IL-2 dual FluoroSpot is described elsewhere (40).

#### Intracellular IL-17A/IL-22 Staining

Cells were cultured as for the cultured IFN-γ/IL-2 FluoroSpot assay. Day 13 T cells were stimulated with antigen loaded monocytes at a 10:1 T cell:monocyte ratio for 19 h, with the addition of Brefeldin A at 10 µg/ml for the last 15 h. Extra- and intra-cellular staining was performed as described previously (34).

### Complement Lysis

CellTrace Violet stained PBMC were stained with anti-bovine CD4 mAb ILA12 or with isotype control Av37 at 3 µg/ml in cell culture medium for 30 min at RT. Next, freshly prepared and sterile filtered baby rabbit serum (Bio-Rad) was added to a final concentration of 10% and cells incubated at 37°C 5% CO_2_ for 60 min with mixing of the cells every 15 min. Cells were washed twice with cell culture medium to remove the baby rabbit serum and cell debris, and were incubated with antigen as described.

### Data Analyses and Statistics

Cytokine concentrations were calculated by interpolating from recombinant cytokine standard curves using a four-parameter sigmoidal function for non-linear regression curve fitting in Prism 7 (GraphPad Software). Descriptive and statistical analyses were performed in Prism or Excel (Microsoft). Where appropriate medium control values were subtracted from the antigen stimulation values and this is indicated as Δ (delta) response variable. Means are shown in all graphs analyzed, except when data were analyzed with a non-parametric test in which case the median is shown. Error bars indicate SEM. The number of spot forming colonies (SFC) per 10^3^ input cells, was calculated according to [S × (N/E)]/IR, where Input cell ratio (IR) is the number of input cells divided by 10^3^; N is the number of harvested T cells on day 13; E is the number of cells/well in the FluoroSpot and S is the number of SFC/well, as described by Vordermeier and Whelan for cultured ELISPOT (41). Where data is representative for multiple experiments this is indicated in the figure legends, otherwise data represent a single experiment.

Unpaired data were compared using simple T-tests, whereas paired data were compared by (one or two-way) repeated measured ANOVA to account for the pairing across multiple groups (or paired T test when only two groups were compared). Except for the antigen presentation blocking data, since the paired difference following blocking is proportional to baseline values and therefore a ratio paired T-test was used. In all cases where multiple comparisons were made within the same graph, p-values were corrected using the Holm-Šídák method, except for Friedman test which is a non-parametric test and was followed by Dunn’s method for multiple comparisons. Comparison of proliferation between the three groups was analyzed using unpaired two-sided T-tests with Welch correction for unequal variance, whereas proliferation between T cell subsets within animals was compared using repeated measures one-way ANOVA. Cytokine concentrations were compared to the control animals using unpaired two-sided T-tests with Welch correction for unequal variance. The effect of CD4 depletion or mock depletion was compared using paired T-tests. The effects of Proteinase K and 0.22 M sodium hydroxide treatment was compared to the respective control treatments using paired T-tests. Activation of expanded T cells and intracellular cytokine staining following re-stimulation was compared using two-way repeated measures ANOVA followed by *post-hoc* tests to compare antigen stimulation between CMEbcg and PPDB expanded T cell populations and compare antigen stimulation to medium control within T cell populations, respectively. Specific lysis of monocytes was compared between antigens using a repeated measures one-way ANOVA. Cultured FluoroSpot cytokine responses were compared using Friedman test, followed by post-hoc comparison of antigen stimulation to medium control. Correlations between total pathology score and immune parameters were analyzed by Pearson correlation. P-values corrected for multiple comparisons ≤0.05 were considered significant. Corrected p-values between 0.05> and ≤0.1 were considered a trend and are indicated in the figures. *p ≤ 0.05, **p ≤ 0.01, ***p ≤ 0.001, ****p ≤ 0.0001.

## Results

### A Hydrophobic Extract of BCG Contains a Diverse Repertoire of Lipids

**Figure 1:**
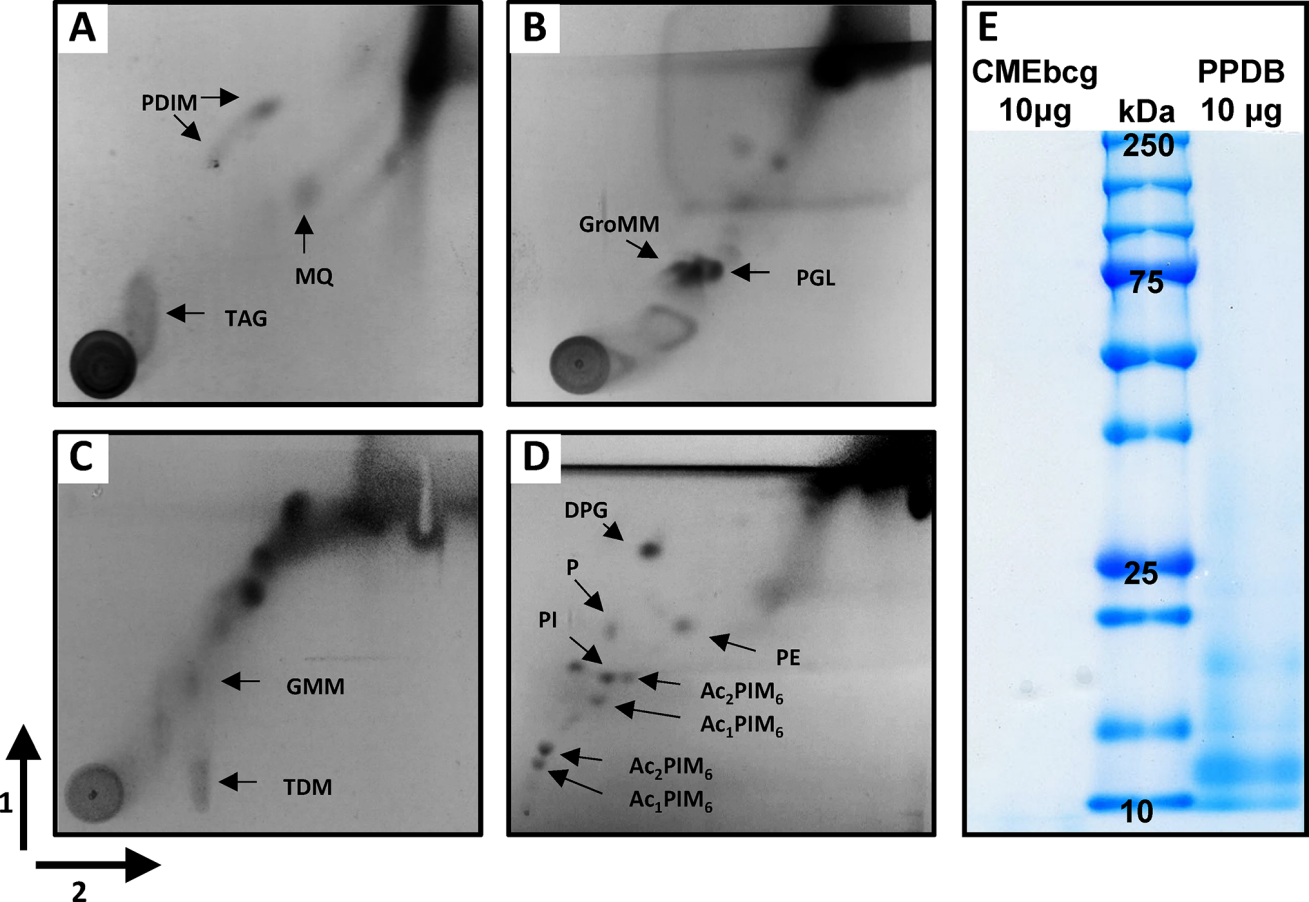
Characterization of a chloroform-methanol extract of *Mycobacterium bovis* BCG. **(A)** Analysis of lipid content of CMEbcg by 2D-TLC followed by molybdophosphoric acid staining using system A. **(B)** system C. **(C)** system D. **(D)** System E. **(E)** Comparison of protein content of CMEbcg and PPDB by SDS-PAGE followed by Coomassie staining. PDIM, apolar phthiocerol dimycocerosates; MQ, menaquinone; TAG, triacyl glycerol; GroMM, glycerol monomycolate; PGL, phenolic glycolipid; TDM, trehalose dimycolate; GMM, glucose-monomycolate; DPG, diphosphatidyl glycerol; P, phospholipid; PE, phenylethanolamine; PI, phosphatidylinositol. Several mono- and di-acylated phosphatidylinositol mannosides (PIMs). 1 and 2—running direction TLC.

Hydrophobic antigens were extracted from the attenuated *M. bovis* strain BCG by chloroform-methanol extraction, followed by a 0.9% NaCl wash to remove any hydrophilic (protein) contaminants (42). To determine the composition of this chloroform-methanol extract of BCG (CMEbcg), the lipid content was characterized by 2D-TLC (**Figures 1A–D**). Diverse (glyco)lipids were identified in CMEbcg, including lipids that are known T cell antigens in cattle, i.e. phosphatidylinositol mannosides (30) and glucose-monomycolate (29). The distribution of different lipid classes was highly comparable to previously published 2D-TLC characterizations of chloroform-methanol extracts of *M. bovis* (36). To determine whether proteins were present in CMEbcg, we performed SDS-PAGE followed by Coomassie staining (**Figure 1E**). The results were compared to purified protein derivative of *M. bovis* (PPDB), a crude protein extract that is the most widely used antigen extract in TB research and diagnostics (43). There were no visible protein bands between 10 and 250 kDa for CMEbcg, whereas there was clear staining of proteins for PPDB. The data presented in **Figure 1** confirm that CMEbcg contains a diverse repertoire of (glyco)lipids and no detectable proteins.

### Hydrophobic Antigens Elicit T Cell Responses *Ex Vivo* in BCG-Vaccinated and *M. bovis-*Infected Cattle

**Figure 2:**
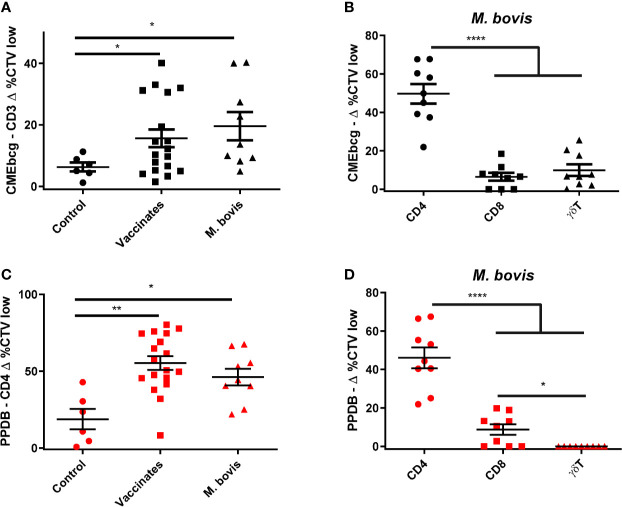
T cell responses against a hydrophobic antigen extract of BCG. CellTrace Violet labeled PBMC isolated from control cattle (Control, n = 6), cattle 6 weeks post BCG vaccination (Vaccinates, n = 18), and cattle infected with *M. bovis* (*M. bovis*, n = 9) were stimulated with CMEbcg (black) or PPDB (red). After 5 days cells were acquired by flow cytometry to measure proliferation as the fraction of CellTrace Violet (CTV) low cells. **(A)** CD3 T cell proliferation following stimulation with CMEbcg. **(B, D)** Proliferation of different T cell populations in *M. bovis* infected animals following stimulation with CMEbcg **(B)** or PPDB **(D)**. **(C)** CD4 T cell proliferation following PPDB stimulation. Mean ± SEM is indicated. Unpaired two-sided T-tests with Welch correction **(A, C)** and repeated measures one-way ANOVA **(B, D)**, followed by Holm-Šídák correction for multiple comparisons.

Since T cells are considered critical in protecting against mycobacterial infections (3, 44) we addressed whether CMEbcg elicits T cell responses in *M. bovis-*immune animals. Peripheral blood mononuclear cells isolated from cows vaccinated with BCG or naturally infected with *M. bovis* were stimulated with CMEbcg or PPDB. Compared to control animals, there was significant T cell proliferation in response to CMEbcg stimulation both in BCG-vaccinated and in *M. bovis-*infected animals (**Figure 2A**). Comparison of proliferation of CD4, CD8, and γδ T cells from infected animals showed that proliferation was significantly higher for CD4 T cells than for CD8 and γδ T cells (**Figure 2B**). Following PPDB stimulation, proliferation within the total CD3 population was not distinct between groups (data not shown), but proliferation of CD4 T cells was significantly higher for BCG-vaccinated and *M. bovis-*infected animals (**Figure 2C**). Proliferation of CD4, CD8, and γδ T cells from *M. bovis-*infected animals was similar to CMEbcg stimulation, with CD4 T cells showing the strongest response (**Figure 2D**). Interestingly, CD4 T cell proliferation following CMEbcg and PPDB stimulation was comparable in *M. bovis* infected animals, with averages of 50 and 46% CTV low CD4 T cells, respectively.

**Figure 3:**
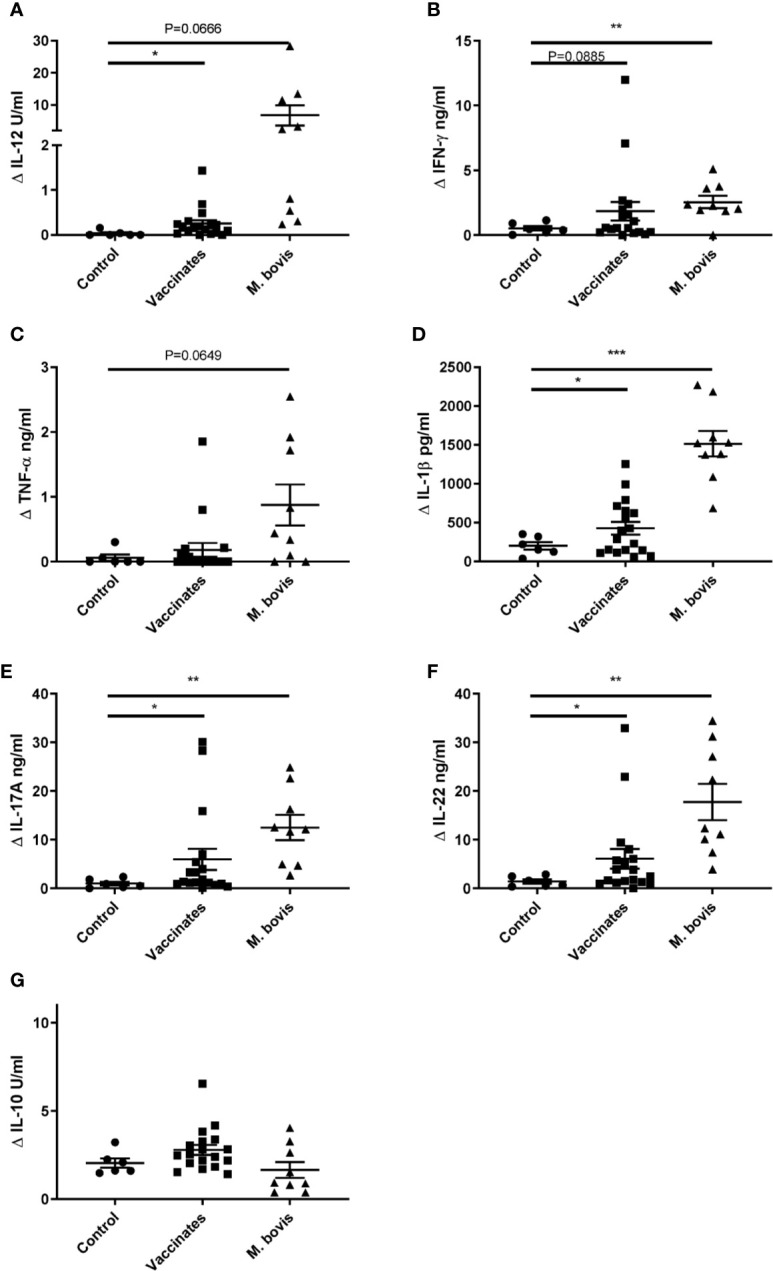
Hydrophobic BCG antigens elicit strong cytokine responses. CellTrace Violet labelled PBMC isolated from control cattle (Control, n = 6), cattle 6 weeks post BCG vaccination (Vaccinates, n = 18) and cattle infected with *M. bovis* (*M. bovis*, n = 9) were stimulated with CMEbcg. Supernatant was harvested after 5 days to measure IL-12 **(A)**, IFN-γ **(B)**, TNF-α **(C)**, IL-1β **(D)**, IL-17A **(E)**, IL-22 **(F)**, IL-10 **(G)** cytokine production by ELISA. Mean ± SEM is indicated. Unpaired two-sided T-tests with Welch correction, followed by Holm-Šídák correction for multiple comparisons.

To further characterize the immune response elicited by CMEbcg, we determined cytokine secretion following CMEbcg stimulation of PBMC (**Figures 3A–G**). Compared to control animals, the release of the Th1 associated cytokines IL-12 and IFN-γ tended to be higher or were significantly higher for vaccinated and *M. bovis* infected animals. The release of the pro-inflammatory cytokine IL-1β was significantly higher in both the BCG vaccinated as well as the *M. bovis* infected animals, whereas TNF-α tended to be higher in the *M. bovis* infected animals. Interleukin-17A and IL-22, the archetypic cytokines for Th17 (/Th22) type immune responses, were significantly higher in both the vaccinates and the infected animals. In contrast, IL-10 release, associated with Th2 and immune regulation, showed no differences between the groups. The cytokine release profile following PPDB stimulation was comparable to CMEbcg stimulation (**Figures S2A–G**). Cytokine responses following CMEbcg stimulation were higher in *M. bovis* infected than in BCG vaccinated animals, except for IL-10, and this was also seen following PPDB stimulation. Whereas BCG vaccination leads to a local infection, *M. bovis* infection is more widespread and the stronger immune response in *M. bovis* infected animals may be the result of both stronger innate stimulation and antigen exposure. To further characterize the role of CD4, CD8, and γδ T cell subsets in the immune response against CMEbcg, CMEbcg stimulation was compared following CD4 T cell depletion of PBMC. Depletion of CD4 T cells significantly abrogated both T cell proliferation and IFN-γ responses, where the reduction was proportional to the level of CD4 depletion, confirming the importance of CD4 T cells in the immune response against CMEbcg (**Figure S2H**).

Summarizing these results, CMEbcg elicits T cell responses with a Th1 and Th17 cytokine release profile that has been associated with protection against bovine tuberculosis in previous studies (45–48).

### Lipopeptides Are the Immunodominant Antigens in CMEbcg

**Figure 4:**
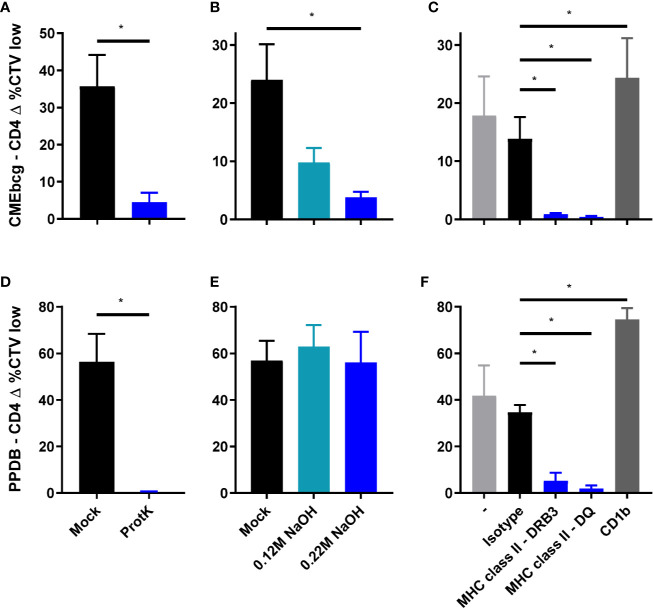
Characterization of the immunodominant antigens in CMEbcg. PBMC from *M. bovis* infected animals (n = 4) **(A, B, D, E)** or BCG vaccinated animals (n = 4) **(C, F)** were stimulated with normal, mock, or enzymatically/chemically treated CMEbcg at 20 µg/ml **(A–C)** or PPDB at 300 IU/ml **(D–F)**. After 5 days CD4 T cell proliferation was measured as the percentage of CellTrace Violet low cells. **(A, D)** Proteinaceous antigens were digested with Proteinase K. **(B, E)** Antigens were delipidated by sodium hydroxide treatment. **(C, F)** PBMC were preincubated with blocking monoclonal antibodies against MHC class II (DRB3 and DQ) or CD1b at 10 µg/ml. Mean ± SEM is indicated. All data are representative for two separate experiments. Paired T-test **(A, B, D, E)**. Ratio paired T-test, followed by Holm-Šídák correction for multiple comparisons **(C, F)**.

Having assessed the biochemical composition of CMEbcg and verified its immunogenicity, we next sought to determine the biochemical nature of the immunogenic fractions. CMEbcg and PPDB were treated with proteinase K or sodium hydroxide to degrade all proteins/peptides or lipids, respectively, and the effect of these treatments on T cell stimulation was assessed. Degrading proteinaceous antigen components abrogated CD4 T cell proliferation following both CMEbcg and PPDB stimulation (**Figures 4A, D**). This was expected for the crude protein extract PPDB, but was surprising for CMEbcg as this extract contains various antigenic lipids, but has a very low protein content. However, delipidation also abrogated the immunogenicity of CMEbcg (**Figure 4B**). As expected, delipidation had no effect on the immunogenicity of PPDB (**Figure 4E**). The effects of protein degradation and delipidation on the stimulation of other T cell subsets was comparable to the effects on CD4 T cell stimulation (**Figure S3**). In humans, lipopeptides have been identified as the immunodominant antigens in hydrophobic extracts of *M. tuberculosis* (27, 28). The above results show that both protein/peptide and lipid components present in CMEbcg are important for its immunogenicity, indicating that lipopeptides are also the immunodominant antigens in CMEbcg for *M. bovis* immune cattle.

Lipopeptides contain protein as well as lipid domains that could potentially be presented to T cells *via* classical MHC and CD1, respectively. The role of these different antigen presenting molecules in presenting antigens to CMEbcg-specific T cells was assessed by testing blocking by antibodies specific for MHC class II and CD1b. Blocking MHC class II presentation almost completely abolished CD4 T cell stimulation by CMEbcg, whereas blocking of CD1b had no blocking effect (**Figure 4C**). Blocking of MHC class II presentation also abrogated CD4 T cell responses against PPDB (**Figure 4F**). Whereas individual T cells will be restricted to a single antigen presenting molecule, blocking of either DRB or DQ almost completely inhibited T cell activation, both for PPDB and CMEbcg. This is a common finding when blocking antigen presentation in polyclonal cell lines (27, 28, 49–51). These blocking experiments showed that lipopeptides in CMEbcg are presented to CD4 T cells *via* MHC class II.

### Antigen-Specific Expansion of Polyfunctional T Cells Following CMEbcg Stimulation

**Figure 5:**
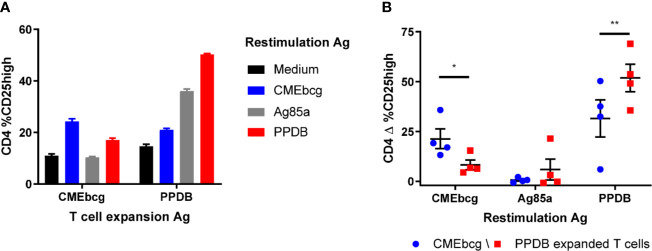
Preferential activation of expanded T cells by cognate antigens. Short term T cell cultures were set up by stimulating PBMC from BCG vaccinated animals (n = 4) with CMEbcg or PPDB. On day 10, expanded T cells were re-stimulated with CMEbcg, Ag85a, or PPDB and autologous *Theileria annulata* cells as antigen presenting cells. On day 13 T cell activation was measured as percentage of CD25 high cells. Comparison of antigen-specific T cell activation of CMEbcg or PPDB expanded T cells for a single animal **(A)** and between four animals **(B)**. Mean ± SEM is indicated. Data are representative for two separate experiments. Two-way repeated measures ANOVA followed by Holm-Šídák correction for multiple comparisons of antigen stimulation between CMEbcg and PPDB expanded T cell populations **(B)**.

Potential novel vaccine antigens have to induce antigen-specific memory. Stimulation of PBMC directly *ex vivo* predominantly detects activation of effector T cells, whereas re-stimulation of cultured (antigen expanded) T cells allows interrogation of the memory T cells response (41, 52, 53). To confirm that T cell expansion and activation in response to CMEbcg is antigen-specific, we established short term T cell cultures using either CMEbcg or PPDB for primary T cell expansion. Expanded T cells were re-stimulated with CMEbcg, PPDB, and Ag85a, using CD25 expression as a marker to measure T cell activation (**Figure 5**). In CMEbcg expanded T cells CD25 upregulation was highest following CMEbcg re-stimulation, followed by PPDB re-stimulation, whilst Ag85a re-stimulation was no higher than observed in the unstimulated (media only) control (**Figures 5A, B**). Conversely, re-stimulation of the PPDB expanded T cell lines with PPDB resulted in the highest level of CD25 expression. The preferential activation of expanded T cells following stimulation with their cognate antigens demonstrates that CMEbcg and PPDB have different antigenic composition and that the T cell expansion observed is antigen-specific. The lower level of T cell stimulation observed with the reciprocal antigen extracts is most likely due to non-proteinaceous antigens within the PPDB extract. Proteomic analysis has shown that lipopeptides are present in PPDB (54, 55), implying that there is a degree of commonality in non-protein antigens. However, the substantial disparity in the responses induced by Ag85a stimulation of the CMEbcg and PPDB expanded lines from an Ag85a-responding animal confirms critical differences in the antigenic specificity of responses elicited by CMEbcg and PPDB (**Figure 5A**).

**Figure 6:**
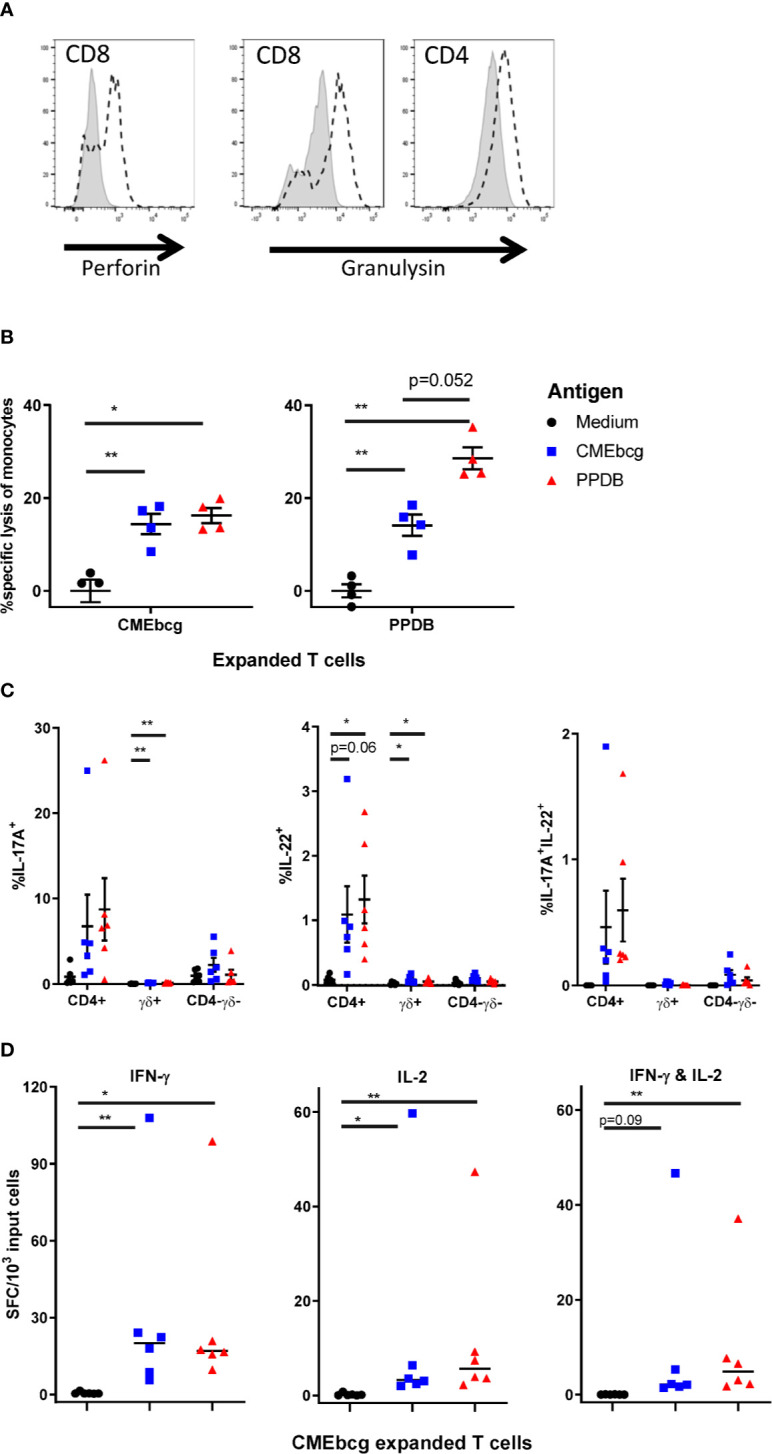
Characterization of CMEbcg and PPDB expanded T cells. PBMC from BCG vaccinated animals were stimulated with CMEbcg or PPDB and cultured for 13 days. **(A)** Perforin and granulysin expression measured by flow cytometry on day 13 post CMEbcg stimulation. Isotype control, shaded area. Perforin/Granulysin staining, dashed line. **(B)** Killing of antigen loaded monocytes by antigen expanded T cells (n = 4). CellTrace Violet stained monocytes were cultured with antigen expanded T cells at an effector:target ratio of 20:1 with or without antigen. After a 20 h incubation period, monocyte viability was measured by flow cytometry. Data are representative for two separate experiments. **(C)** Intracellular cytokine staining for IL-17A and IL-22, 19 h after re-stimulation of CMEbcg expanded T cells (n = 6) with antigen loaded monocytes. Cells were co-stained for CD4 and gamma delta T cell (γδ) markers. **(D)** Cultured IFN-γ and IL-2 FluoroSpot of CMEbcg expanded T cells (n = 6) stimulated with antigen loaded monocytes for 20–22 h. Numbers of total IFN-γ, IL-2 (single and dual secreting), or dual IFN-γ/IL-2 secreting cells are shown. Mean ± SEM is indicated in **(B, C)**, median in **(D)**. Repeated measures one-way ANOVA followed by Holm-Šídák correction for multiple comparisons **(A)**. Two-way repeated measures ANOVA followed by Holm-Šídák correction for multiple comparisons of antigen stimulation to medium control within T cell populations **(C)**. Friedman test, followed by Dunn’s method for multiple comparisons **(D)**.

The ability of T cells to directly kill and/or inhibit the growth of intracellular *M. bovis* (and of *M. bovis* infected cells) and to produce cytokines have both been associated with immunological protection against bovine tuberculosis (7, 45, 47, 56, 57). To examine the potential functionality of CMEbcg-expanded T cells, we performed a number of assays to describe their functional phenotype. Firstly, we assessed the expression of perforin and granulysin, anti-mycobacterial effector molecules of the cytotoxic pathway (58–60). Following CMEbcg stimulation, perforin expression was upregulated in CD8 T cells and granulysin expression was upregulated in both CD8 and CD4 T cells (**Figure 6A**, **Figure S4**). To explore whether CMEbcg-specific T cells are cytotoxic, killing of autologous antigen-loaded monocytes by expanded T cells was analyzed by flow cytometry (**Figure 6B**). Compared to co-incubation without antigen, loading monocytes with CMEbcg significantly increased the killing of monocytes by CMEbcg expanded T cells. CMEbcg-expanded T cells also killed PPDB loaded monocytes. Similar results were found for PPDB-expanded T cells, where significant increased killing of monocytes was seen both for PPDB and CMEbcg antigen loading, with a tendency for a higher killing of PPDB loaded than CMEbcg loaded monocytes (p = 0.052). Similar to the re-stimulation assays, the ability of CMEbcg and PPDB-expanded T-cell populations to recognize monocytes loaded with the reciprocal extract indicates that the two extracts shared common antigenic components. Antigen loading without the addition of cultured T cells had no effect on monocyte viability (data not shown), excluding a direct cytotoxic effect of the antigen extracts.

We examined the cytokine responses of CMEbcg expanded memory T cells by intracellular IL-17A and IL-22 staining and cultured IFN-γ and IL-2 FluoroSpot (**Figures 6C, D**), focusing on cytokines that have been associated with protection in *M. bovis* vaccination-challenge experiments (45–48, 57). Overall two-way repeated measures ANOVA indicated that there was a significant effect of re-stimulation of CMEbcg-expanded T cells with PPDB or CMEbcg on the number of IL-17A (p = 0.046) and IL-22 positive cells (p = 0.012) (**Figure 6C**). Looking at the different T cell populations, the percentage of positive cells was highest within CD4 T cells and was slightly higher following PPDB re-stimulation compared to CMEbcg. Total IL-17A^+^IL-22^+^ cells were significantly higher following CMEbcg (p = 0.019) and PPDB (p = 0.019) restimulation (Friedman test followed by Dunn’s method for multiple comparisons) and polyfunctional IL-17A^+^IL-22^+^ cells were predominantly CD4 T cells (**Figure 6C**). The number of IFN-γ secreting cells, measured by FluoroSpot, was significantly higher following CMEbcg and PPDB re-stimulation of CMEbcg-expanded T cells (**Figure 6D**). For IL-2, there was a significant increase in the number of secreting cells following both CMEbcg or PPDB re-stimulation. Almost all IL-2-positive secreting cells were polyfunctional, also secreting IFN-γ (**Figure 6D**). As a comparison and control, we also looked at the cytokine responses of PPDB-expanded T cells. As expected, PPDB-expanded cultured T cells also produced IL-17A, IL-22, IFN-γ, and IL-2 (**Figure S5**). For IL-2, IFN-γ, and IL17A the fraction of positive cells was higher following re-stimulation of PPDB-expanded T cells compared to CMEbcg-expanded T cells. Notably, the opposite was the case for IL-22^+^ and IL-17A^+^IL-22^+^ cells, where the fraction was higher in the CMEbcg expanded T cells.

Overall, these experiments show that CMEbcg-specific cultured T cells, which are indicative for a central memory T cell pool, have functional characteristics that are associated with protection against *M. bovis* disease.

### CMEbcg-Specific T Cell Proliferation Post BCG Vaccination Was the Best Predictor for Protection Against *M. bovis* Challenge

**Figure 7:**
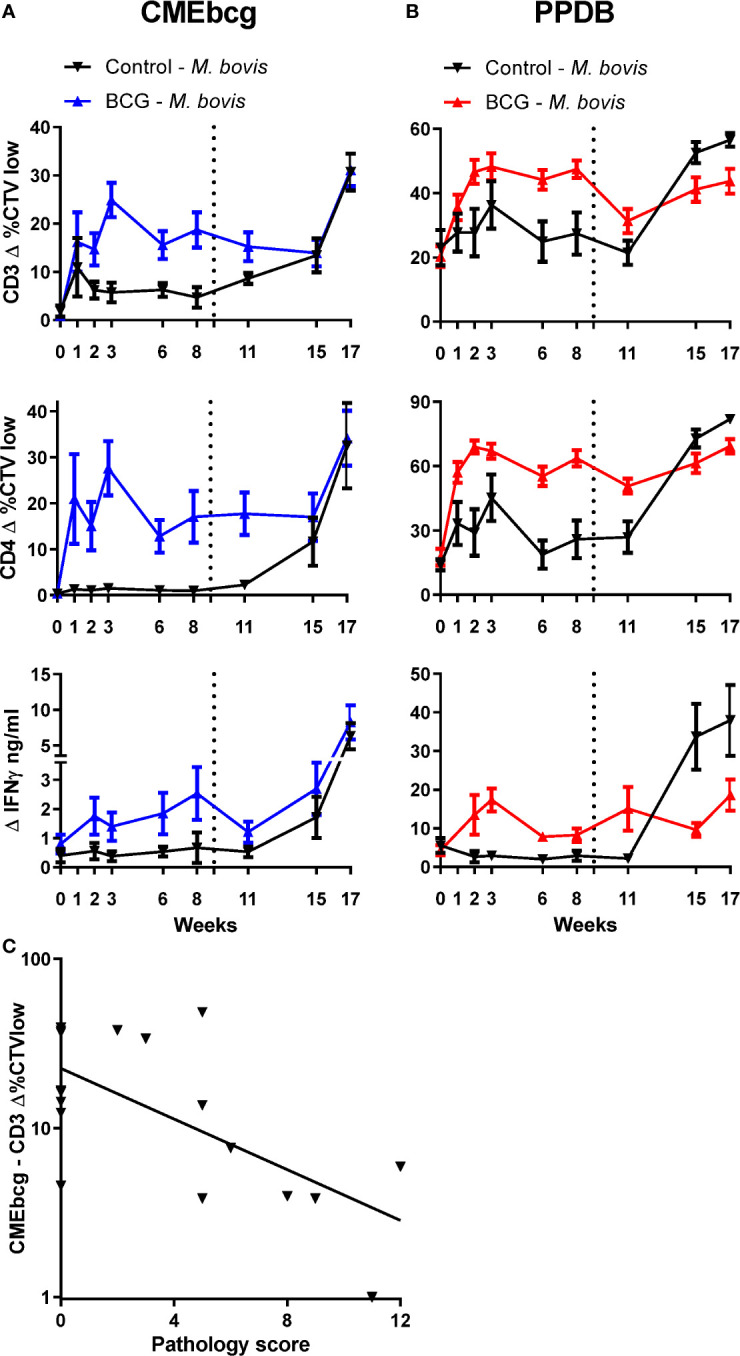
Longitudinal characterization of immune responses against CMEbcg and PPDB in BCG vaccinated and *M. bovis* challenged calves. Six-month-old male Holstein-Frisian cross calves (n = 18) were vaccinated subcutaneously with 4.6 × 10^6^ CFU BCG Danish SSI 1331. Six calves served as controls. In week 9 (dotted line) all animals (n = 24) were challenged endobronchially with 10^4^ CFU *M. bovis* AF2122/97. PBMC were stimulated for 5 days with CMEbcg **(A)** or PPDB **(B)** and proliferation was measured by flow cytometry as the percentage of CellTrace Violet low cells and supernatant was harvested to measure IFN-γ release by ELISA. Mean ± SEM is indicated. **(C)** Correlation between CMEbcg-specific T-cell proliferation 8 weeks post BCG vaccination and gross pathology score 11 weeks after *M. bovis* challenge within BCG vaccinated calves (n = 18). Pathology scores were based on the macroscopic identification of lesions in lymph nodes and lung tissue at post-mortem examination (31).

In order to examine the relevance of immune responses against hydrophobic antigens, CMEbcg-specific T cell responses were measured longitudinally in calves enrolled in a BCG vaccination and *M. bovis* challenge study (**Figure 7**). A week following BCG-vaccination CMEbcg-specific T cell responses could be detected, measured as CD4 T cell proliferation and IFN-γ secretion following *ex vivo* stimulation of PBMC (**Figure 7A**). Total and CD4 T cell proliferation peaked at week 3, whereas IFN-γ responses peaked at 8 weeks post vaccination. In contrast there were no changes in the immune response against CMEbcg in the control animals, except for a transient rise in CD3 T cell proliferation in week 1. Nine weeks after BCG vaccination, both control and vaccinated animals were challenged endobronchially with a virulent strain of *M. bovis*. *M. bovis* challenge induced CMEbcg-specific immune responses in the unvaccinated animals, with responses detectable at 2 weeks post infection and rising until 8 weeks post infection. In the BCG vaccinated animals, challenge boosted CMEbcg-specific immune responses and responses were higher than following vaccination. Longitudinal responses to PPDB (**Figure 7B**) were comparable to those described for CD4 T cells following CMEbcg stimulation. Except for one difference, the CMEbcg-specific responses following challenge were similar for vaccinated and unvaccinated calves at the final timepoint, whereas for PPDB the response was higher in the unvaccinated than the vaccinated animals. Proliferation of CD8 and γδ T cells was comparable to CD4 T cell responses (**Figure S6**). Altogether, both BCG vaccination and *M. bovis* infection rapidly induced a robust immune response against CMEbcg.

Next, we analyzed whether immune responses post BCG vaccination, but before *M. bovis* challenge, correlated with protection against bovine tuberculosis. Protection was defined as a reduction in the severity of *M. bovis* associated lesions in lung and lymph nodes assessed 11 weeks post challenge at post mortem examination. T cell proliferation and IFN-γ release following stimulation with CMEbcg and PPDB were selected as potential indicators of immunity. Ranking the correlations between the BCG-vaccination-induced immune parameters before challenge and the pathology score following *M. bovis* infection showed that only CMEbcg-specific T cell proliferation correlated significantly with challenge outcome (**Table 2**). CMEbcg-specific T cell proliferation showed a significant and negative association with pathology following *M. bovis* challenge (**Figure 7C**). In this vaccination and challenge study, T cell responses against the hydrophobic antigen extract CMEbcg in BCG-vaccinated animals were associated with protection against pathology resulting from *M. bovis* challenge.

**Table 2:** Correlations between immune responses post BCG vaccination and pathology following *M. bovis* challenge.

Immune response post BCG vaccination				
Antigen	Immune parameter	Pearson R	95% C.I.	p-value^1^
CMEbcg	CD3 T cell proliferation	−0.66	−0.86 to −0.27	**0.013**
PPDB	CD3 T cell proliferation	−0.54	−0.80 to −0.09	0.063
CMEbcg	IFN-γ release	−0.43	−0.75 to 0.04	0.141
PPDB	IFN-γ release	−0.14	−0.57 to 0.35	0.590

Pearson correlation between selected immune parameters 8 weeks post BCG vaccination and pathology scores at post mortem 11 weeks after M. bovis challenge within BCG vaccinated calves (n =18). Calves were vaccinated with BCG and challenged with M. bovis as described in **Figure 7**. Immune parameters, shown in **Figure 7**, were log transformed. Pathology scores were based on the macroscopic identification of lesions in lymph nodes and lung tissue at post-mortem examination. ^1^ p-values adjusted for multiple comparisons using the Holm-Šídák method and p-values ≤ 0.05 are in bold.

## Discussion

Additional as well as alternative control strategies are needed to curtail the impact of bovine tuberculosis on animal and human health (1, 2). Here we assessed the potential of hydrophobic mycobacterial antigens as novel vaccine candidates against bovine tuberculosis in cattle. A hydrophobic antigen extract of *M. bovis* BCG elicited strong T cell responses in *M. bovis* immune cattle and lipopeptides were shown to be the immunodominant antigens. Immune responses to hydrophobic mycobacterial antigens post BCG vaccination correlated with protection against experimental *M. bovis* challenge. This study underlines the potential of mycobacterial lipopeptides as targets for further research and candidates for novel subunit vaccines against bovine tuberculosis.

Research on vaccines for *M. bovis* has focused on proteinaceous antigens, despite the fact that mycobacteria possess a thick hydrophobic outer layer consisting of (glyco)lipids, lipoproteins, and hydrophobic proteins that are involved in interaction with the host. We found that CMEbcg, a hydrophobic antigen extract of *M. bovis* BCG, elicited robust T cell responses in cattle following BCG vaccination and following both natural and experimental *M. bovis* infection. Chemical and enzymatic degradation indicated that lipopeptides are the immunodominant antigens in CMEbcg. These findings are in line with studies demonstrating lipopeptides to be the immunodominant hydrophobic antigens in *M. tuberculosis* infected humans (27, 28) as well as in BCG vaccinated guinea pigs (61). The *M. bovis* genome encodes for approximately a hundred lipoproteins shown to be important for bacterial homeostasis as well as virulence, playing a role in pathogen-host interaction and immune modulation (20, 62–64). Disrupting the lipidation process of lipoprotein precursors resulted in attenuation of *M. tuberculosis* *in vitro* and *in vivo* (64) and knockout of individual lipoproteins of *M. bovis* or *M. tuberculosis* can also result in attenuation *in vivo* (65–67).

Post-translational modification of lipoprotein precursors occurs by the sequential action of the three enzymes Lgt, LspA, and Lnt. Following recognition of a conserved lipobox motif in the signal peptide, the prelipoprotein is first diacylated, then the signal sequence is cleaved off and, finally, a third lipid moiety is attached. BCG has been shown to use palmitic and tuberculostearic acid as substrates for acylation (62). Because of the lipid and peptide composition of lipopeptides these antigens could potentially be recognized by CD1-restricted lipid-reactive T cells and by conventional MHC-restricted peptide-reactive T cells. Blocking of MHC class II presentation almost completely abolished T cell activation, showing that recognition of the peptide components by MHC class II restricted T cells is the predominant route for T cell stimulation. Together with the strong proliferative and cytokine responses in CD4 T cells and the effect of CD4 T cell depletion on overall T cell activation following CMEbcg stimulation, these data show that CD4 T cells are the main population responding to lipopeptides. Depletion of CD4 T cells also abrogated CD8 and γδ T cell proliferation, suggesting bystander activation of these populations. However, depleting CD4 T cells also abrogates the secretion of growth factors (e.g. IL-2) and it is unknown whether the observed CD8 and γδ T cell responses are a result of bystander activation or cognate antigen stimulation.

There are only two other studies that examined the immunogenicity of hydrophobic mycobacterial antigens in the context of bovine tuberculosis. Van Rhijn, Nguyen (29) found that stimulation of PBMC from *M. bovis* infected cattle with hydrophobic *M. bovis* antigens elicited T cell proliferation. Although they used a chloroform-methanol extraction method similar to the method described here, protease treatment of the hydrophobic antigen extracts did not affect T cell stimulation and they therefore concluded that lipids were the immunodominant antigens in the hydrophobic antigen extract. This is in contrast to our findings that protein degradation almost completely abrogated T cell activation. Compared to their extraction method, we included a Folch wash at the end of the extraction process to remove any hydrophilic (protein) contaminants (42), making the extraction more stringent. Differences in *M. bovis* growth conditions, the extraction method, and in T cell assays complicate a comparison of both studies. However, our finding that lipopeptides, rather than lipids, are the immunodominant hydrophobic antigens are in line with studies in humans (27, 28) and guinea pigs (61). Pirson, Engel (30) demonstrated that phosphatidylinositol mannosides (PIMs), a class of mycobacterial glycolipids, activated T cells from *M. bovis* infected cattle. CMEbcg contained several lipid antigens, including PIMs, glucose monomycolate (GMM), and glycerol monomycolate (GroMM). The glycolipid GMM was discovered as an immunodominant lipid T cell antigen in cattle infected with *Mycobacterium avium* subspecies *paratuberculosis* (29), whereas GroMM is an immunogenic T cell antigen in human tuberculosis (22). In humans, almost all lipid T cell antigens of *M. tuberculosis*, including GroMM and the PIMs, are presented *via* CD1b or CD1c (68). While cattle do not have CD1c, they do express CD1b (69) and GMM was shown to be presented to T cells *via* CD1b (29). Interestingly, while the assay condition used here have shown to be conducive to study (CD1b mediated) lipid antigen presentation (29, 30), blocking of CD1b did not block T cell stimulation by CMEbcg. Together with the importance of MHC class II presentation and in line with findings in TB infected humans (27, 28), this indicates that although lipid-reactive T cells have been found in cattle they are not as numerous as lipopeptide-reactive T cells.

Stimulation with CMEbcg elicited T cell activation expressly in immune animals and led to expansion of antigen-specific T cells, demonstrating that BCG vaccination induces a robust lipopeptide (CMEbcg) specific T cell memory. Although the immune response to CMEbcg was shown to be antigen-specific, CMEbcg-expanded T cells also strongly responded to PPDB re-stimulation and vice versa for PPDB-expanded T cells, indicating there is antigen overlap between the two extracts. This cross reactivity of CMEbcg-expanded T cells to PPDB was also demonstrated in humans (27) and guinea pigs (61). Mass spectrometry analyses of PPDB identified several lipopeptides (54, 55), also indicating antigen overlap between the extracts. Individual immunogenic lipoproteins of *M. bovis* have previously been identified in cattle, e.g. MPB83 (70), LpqH (71), LpqE and LpqD (72). The protein domain of lipoproteins can be quite large (e.g. 500 amino acids for LpqL), but proteins staining of CMEbcg loaded SDS-PAGE gels did not detect any proteins. This suggests that hydrophobic N-terminal lipidated protein fragments (lipopeptides), rather than full lipoproteins, are eluted into the chloroform-methanol solvent and determine the antigenicity of CMEbcg. Only a single known T cell epitope towards the N-terminus of a lipoprotein of *M. bovis* is registered on the Immune Epitope Database (73), but this is on a total of only seven known epitopes from just two studies in cattle (71, 72). Numerous epitopes towards the N-terminus of lipoproteins of *M. bovis* and *M. tuberculosis* have been identified in other species (61, 74). Lipopeptides were recognized by MHC class II restricted T cells and this leaves the question why delipidation nevertheless abrogated T cell activation. There are several explanations of how lipidation of proteins might influence T cell activation: stimulation of APCs through activation of pattern recognition receptors (20, 75), unmasking or modification of epitopes by the lipidation process (76), improved antigen uptake, processing, and presentation of intact lipopeptides (77–79). The intrinsic adjuvant properties of lipopeptides, potentially leading to improved antigen processing and T cell stimulation, make lipopeptides interesting vaccine candidates. Indeed synthetic lipopeptides have been shown to be highly immunogenic in mice (80) and can confer protection in murine vaccination-*M. tuberculosis* challenge models (81, 82). The only example of vaccination against a lipoprotein in cattle was with a plasmid DNA expressing only the protein part of the lipoprotein MPB83. Although in infected cattle MPB83 is an immunodominant lipoprotein, vaccination with an experimental DNA vaccine encoding the protein part of MPB83 induced limited T cell responses and did not protect against *M. bovis* challenge (83). In contrast, vaccination of mice with recombinant MBT83, the *M. tuberculosis* orthologue of MPB83, conjugated to a lipid moiety did confer protection against *M. tuberculosis* challenge (84). Indicating that lipidation of the protein is important for inducting protective immunity.

In the current study, T cell proliferation, specific to the lipopeptide-containing extract, following BCG vaccination was the best predictor for protection against pathology induced by subsequent *M. bovis* challenge. The immunogenicity of hydrophobic mycobacterial antigens and lipopeptides has been described in other species, but this is the first study identifying lipopeptide-specific immune responses as a correlate of protection against pathology induced by a tuberculosis-causing *Mycobacterium* species. *Ex vivo* immune responses against PPDB can be poor correlates of vaccine efficacy (48, 85, 86), similar to the results shown in this study. Several correlates of vaccine efficacy for bovine tuberculosis have been described in cattle, of which *M. bovis* antigen-specific memory T cell responses are particularly promising [reviewed by Vordermeier, Jones (1)]. We demonstrated that CMEbcg stimulation elicits robust lipopeptide (CMEbcg) specific memory T cells that produce IFN-γ, IL-17A, and IL-22. These Th1 and Th17 associated cytokines are associated with protection against bovine tuberculosis (45–48).

It has been hypothesized that the immunogenicity of lipoproteins (and of other immunodominant antigens) may aid mycobacteria by skewing the host immune response towards a non-protective response or a response that contributes to persistence (61, 87). Additionally, lipoproteins can have immunostimulatory or immunosuppressive effects (20). However the results presented here, i.e. secretion of Th1 and Th17 cytokines and cytotoxic activity of the lipopeptide-specific T cells and their association with protection against pathology following experimental challenge, together with the successful lipoprotein vaccination in murine tuberculosis models (e.g. 84, 88, 89), argue against this hypothesis. Protective immunity following vaccination depends on the target antigens and on the quality of the induced immune response. To further address the potential of lipopeptides as vaccine antigens it is important to elucidate whether immune responses to lipopeptides are causally correlated with protection against *M. bovis* infection and if this is the case, to delineate the importance of antigen *versus* quality of the immune response. That is, whether immunity against specific lipoproteins is important and hampers the pathogenesis of *M. bovis* or whether unique immunogenic properties of lipoproteins or BCG infection induces protective antimycobacterial immunity resulting in protection against disease. Detailed characterization of immune responses to individual lipopeptide antigens will form a first step in addressing these questions.

## Conclusion

Lipopeptides of *M. bovis* are highly immunogenic in cattle, inducing robust memory T cell responses with antimycobacterial phenotypes, including secretion of key Th1 and Th17 associated cytokines. This is the first study showing that immune responses to hydrophobic mycobacterial antigens post BCG vaccination correlate with protection against experimental *M. bovis* challenge, emphasizing the potential of mycobacterial lipopeptides as targets for novel subunit vaccines against bovine tuberculosis.

## Data Availability Statement

The raw data supporting the conclusions of this article will be made available by the authors, without undue reservation.

## Ethics Statement

The animal study was reviewed and approved by the Animal and Plant Health Agency Animal Welfare and Ethical Review Body committee and all animal experiments were conducted within the limits of a United Kingdom Home office license under the Animal (Scientific Procedures) Act 1986.

## Author Contributions

TC, MV, and IM acquired funding. TC, MV, IM, DB, and LB contributed to the design of the study. LB, SS, TH, and CV performed the experiments. LB prepared the manuscript. All authors contributed to the article and approved the submitted version.

## Funding

This work was funded by the UK Biotechnology and Biological Sciences Research Council (BBSRC, Grant number BB N004647/1) and the bovine tuberculosis research budget held and administered centrally by the UK Department for Environment, Food and Rural affairs on behalf of England, Scotland and Wales (Project Code SE3299). Work at the Roslin Institute is supported by Strategic Program Grants from the BBSRC.

## Conflict of Interest

The authors declare that the research was conducted in the absence of any commercial or financial relationships that could be construed as a potential conflict of interest.
