# Supplementary figures and images for: Hydrophobic Mycobacterial Antigens Elicit Polyfunctional T Cells in Mycobacterium bovis Immunized Cattle: Association With Protection Against Challenge?
Source: Front Immunol. 2020 Nov 12;11:588180. doi: 10.3389/fimmu.2020.588180 (PMC7688591; doi:10.3389/fimmu.2020.588180)

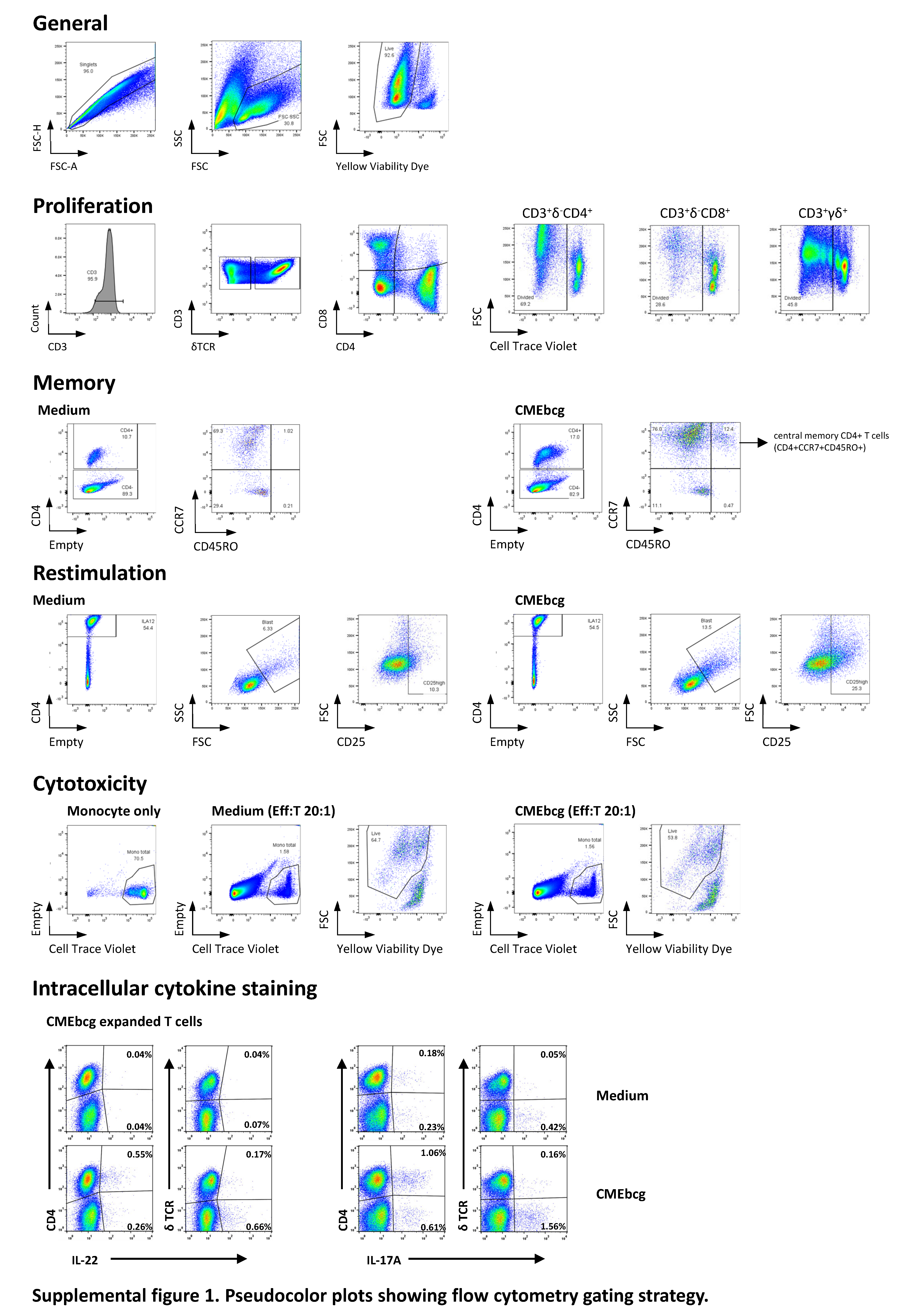

Supplement: Supplementary file 1 [file Image_1.tiff]

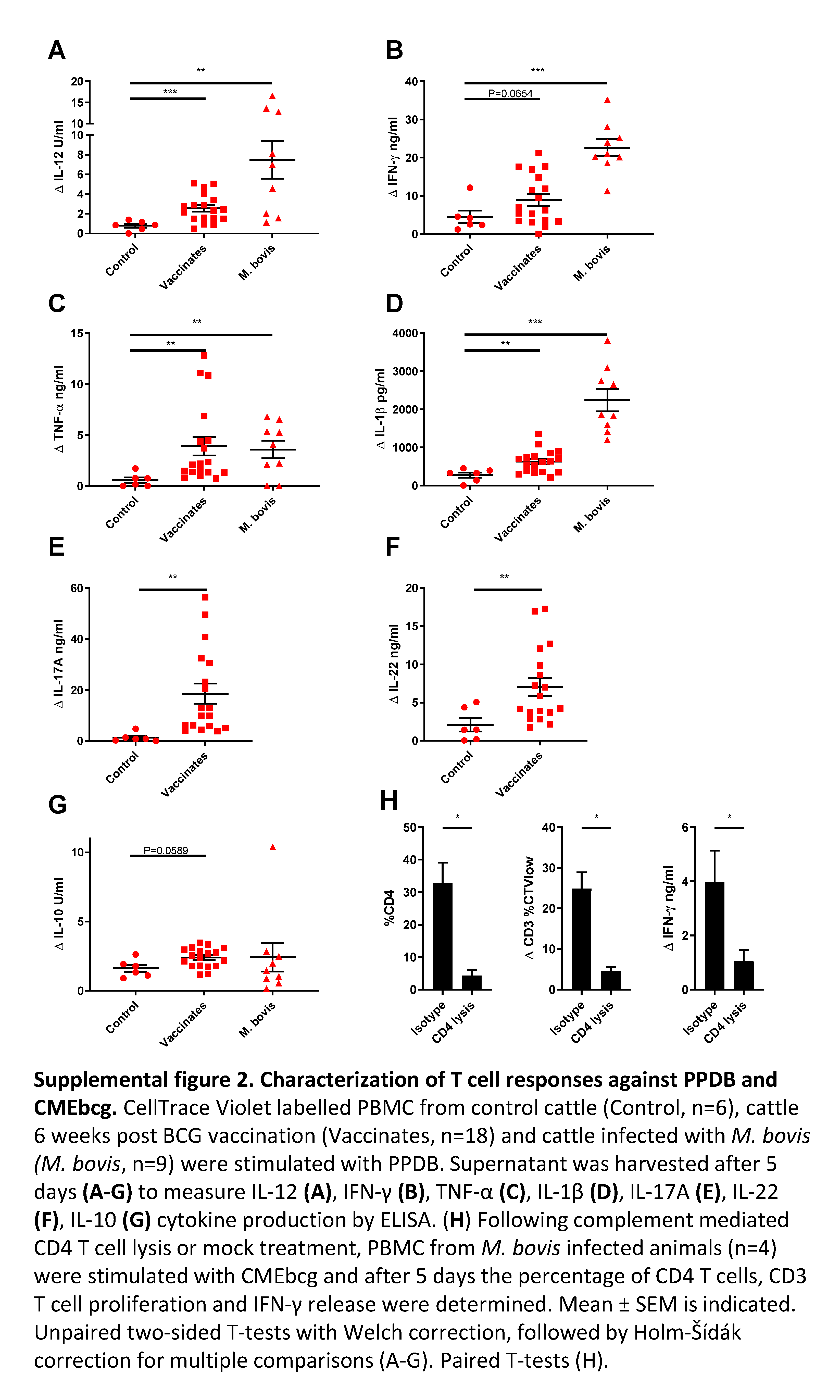

Supplement: Supplementary file 2 [file Image_2.tiff]

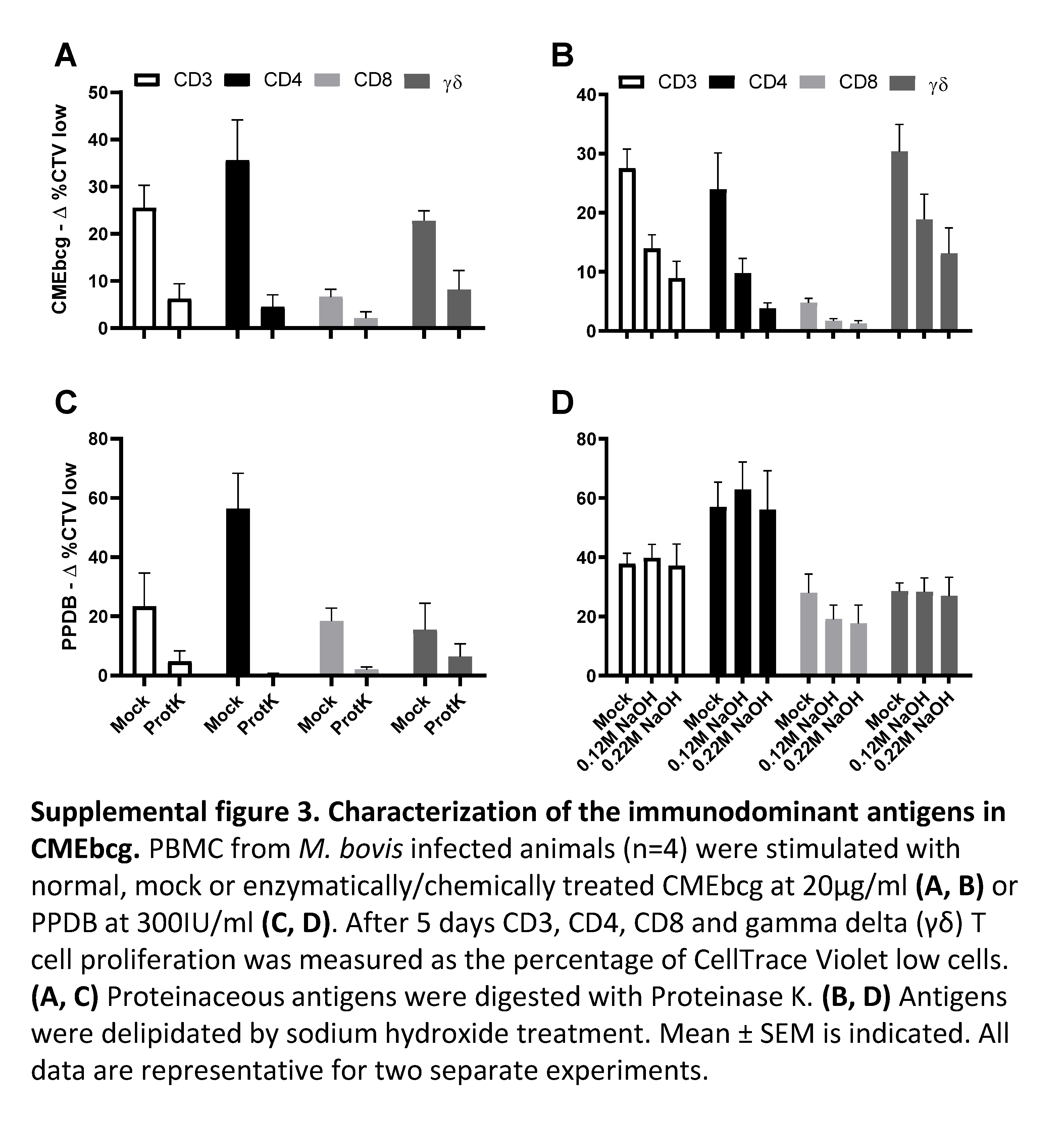

Supplement: Supplementary file 3 [file Image_3.tiff]

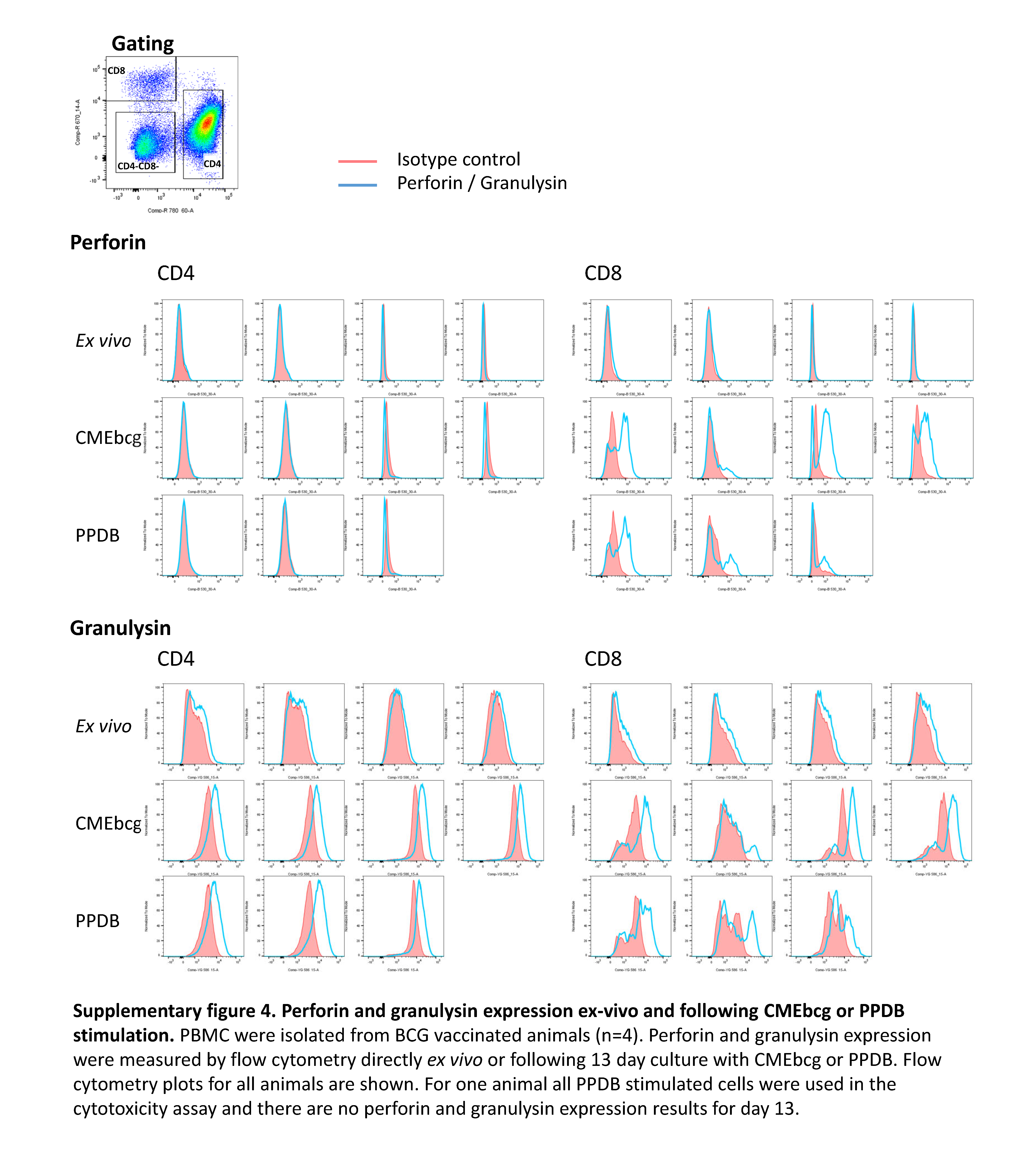

Supplement: Supplementary file 4 [file Image_4.tiff]

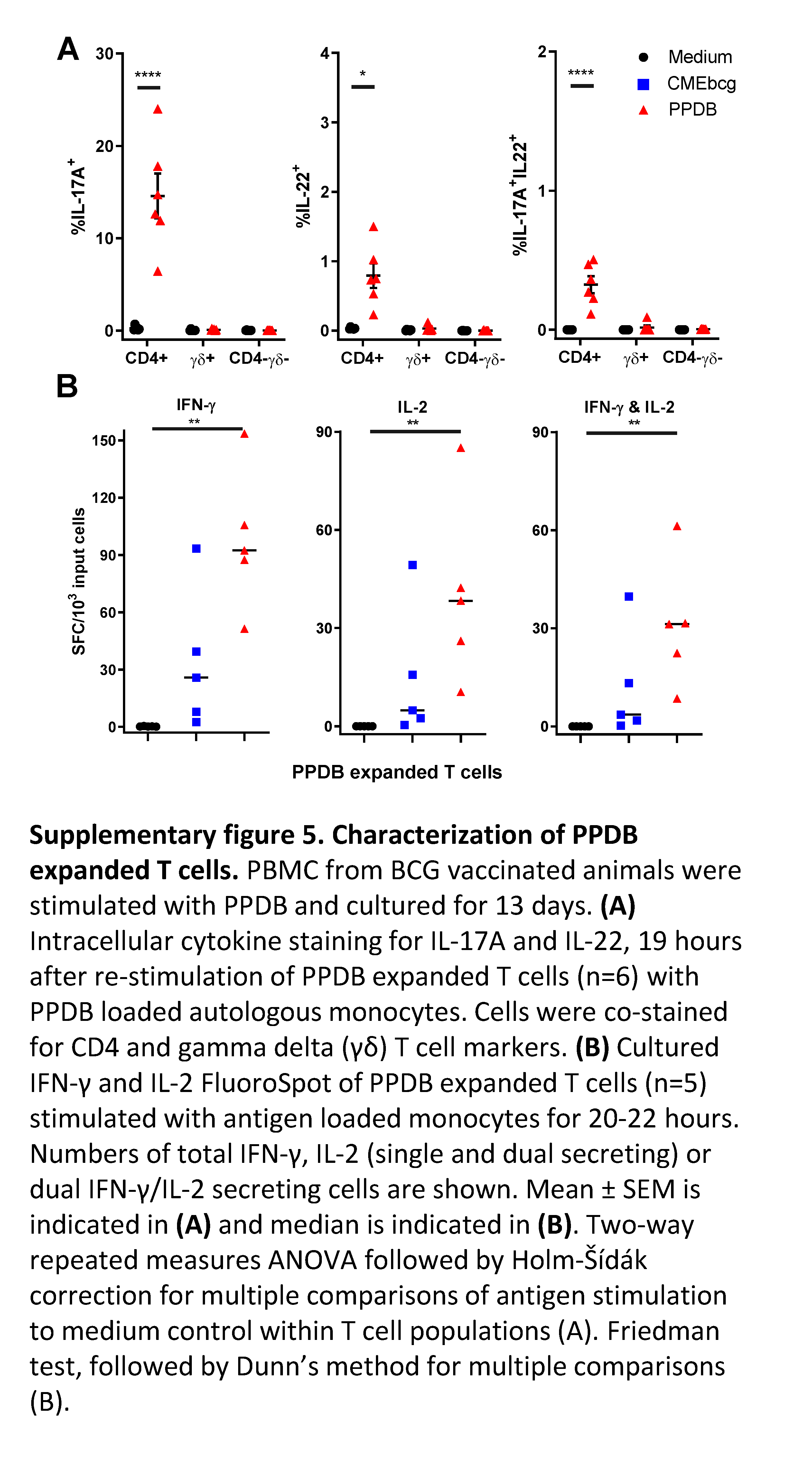

Supplement: Supplementary file 5 [file Image_5.tiff]

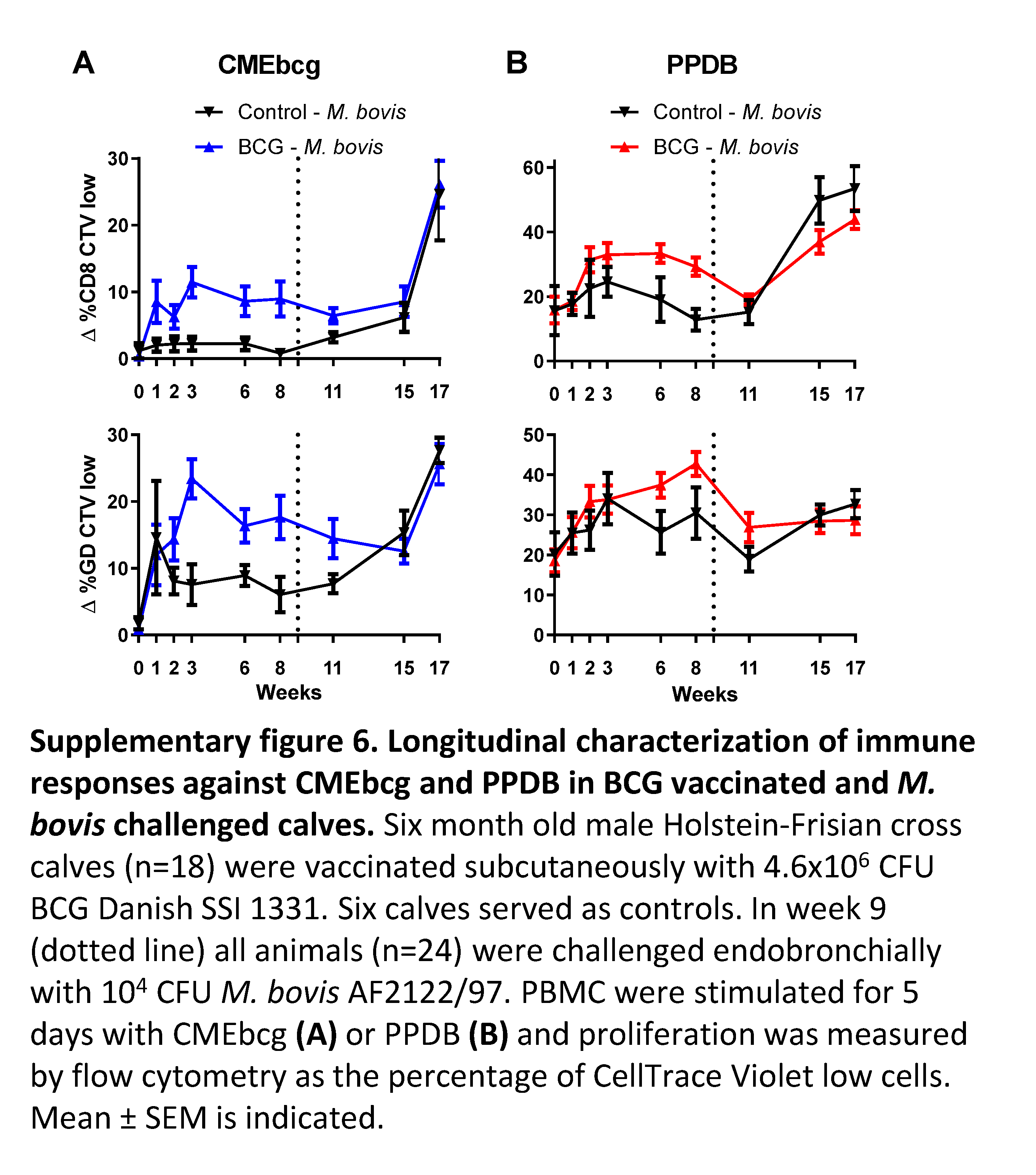

Supplement: Supplementary file 6 [file Image_6.tiff]
